# A Case of Acute Focal Bacterial Nephritis With Negative Pyuria and Urine Culture Test Results

**DOI:** 10.7759/cureus.32942

**Published:** 2022-12-25

**Authors:** Yuji Kaneko, Hiroki Isono

**Affiliations:** 1 Department of Medicine, Hokkaido University School of Medicine, Sapporo, JPN; 2 Department of General Medicine, HITO Medical Center, Ehime, JPN

**Keywords:** sepsis, flank pain, lower back pain (lbp), renal abscess, urinary track infection

## Abstract

Acute focal bacterial nephritis (AFBN) is a radiologically diagnosed acute localized kidney infection that appears in the continuum between a perinephric abscess and renal abscess. We report an unusual case of AFBN presenting without pyuria or positive urine cultures. A 42-year-old woman with chronic lower back pain who regularly used nonsteroidal anti-inflammatory drugs was admitted to our hospital with right-sided abdominal distention, fever, chills, and pain extending from the right lower abdomen to the back since two days. The physical examination revealed no abdominal or costovertebral angle tenderness. Urinalysis was negative. Abdominal ultrasound was notable for an indistinct nodular shadow (32 × 25 mm) on the upper pole of the right kidney. Abdominal contrast-enhanced computed tomography revealed a wedge-shaped area with a minimal uptake of the contrast in the right kidney. The patient was admitted to the hospital, and antimicrobial therapy was started for AFBN. Antibiotics were administered intravenously for one week and orally for two weeks. No relapse of symptoms was observed during the four-month follow-up period. This case report suggests the importance of considering AFBN as a differential diagnosis for cases of idiopathic fever and lateral pain or back pain, even when pyuria and urine culture test results are negative.

## Introduction

Acute focal bacterial nephritis (AFBN) refers to a localized mass lesion without liquefaction in the renal parenchyma caused by bacterial infection. Pathologically, it is characterized by edema of the interstitial tissue and infiltration of inflammatory cells. AFBN was first described by Rosenfield et al. in 1979 [1]. A systematic review revealed that 138 cases of AFBN in adults were reported worldwide from 1979 to 2015; however, the available information consisted mainly of case reports and small case series [2].

The pathophysiology of AFBN remains unclear. One report describes the disease as an intermediate between acute pyelonephritis and progression to a renal abscess [3]. Urinalysis is useful in the diagnosis of acute pyelonephritis. A urinary leukocyte count of 5 white blood cells (WBCs)/high-power field (hpf) or greater has a sensitivity of 72%-95% and specificity of 48%-82% for the diagnosis of acute pyelonephritis; similarly, a Gram staining result of 1 bacterium/hpf or greater has a sensitivity of 93% and specificity of 95% [4]. If there is no history of antibiotic administration and no obstructive origin, pyuria and bacteriuria are almost always present. If AFBN develops from acute pyelonephritis, pyuria and bacteriuria are also expected. Contrary to this, we report a case of AFBN with negative pyuria and urine cultures.

## Case presentation

**Figure 1:**
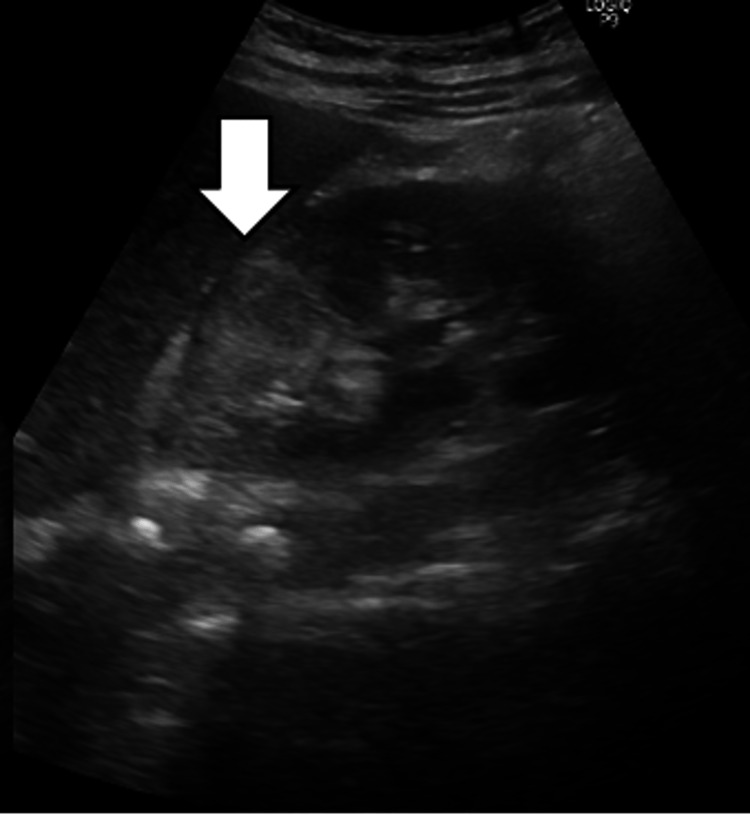
Abdominal ultrasonography showing a nodular shadow (diameter, 32 × 25 mm) on the upper pole of the right kidney

**Figure 2:**
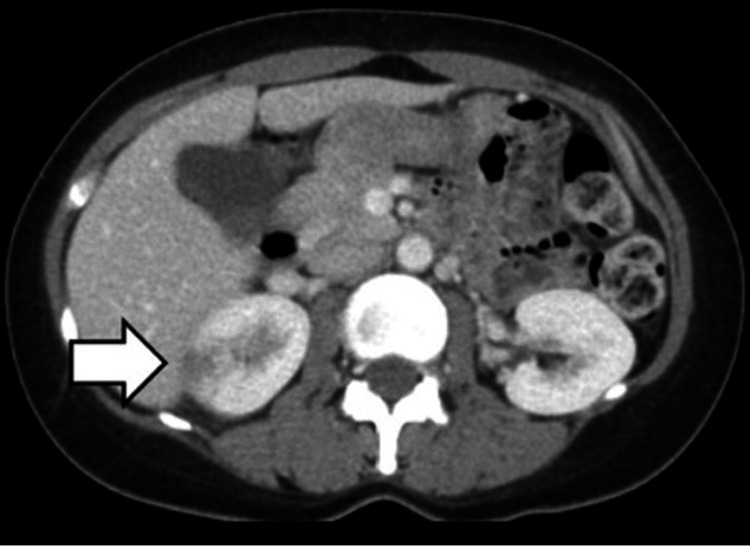
A contrast-enhanced computed tomography image showing a wedge-shaped, partially contrast-impaired area in the right kidney

A 42-year-old woman presented to the emergency department with a two-day history of fever (39℃), chills, loss of appetite, right-sided abdominal distention, and pain extending from the right side of the abdomen to the back. No signs of cystitis, such as dysuria, frequent or urgent urination, or hematuria, were observed. She had undergone discectomy for a herniated lumbar L4/L5 disc six years prior to presentation. Her current medications included pregabalin, etodolac, rebamipide, and etizolam. She was regularly using nonsteroidal anti-inflammatory drugs due to severe postoperative pain. Her temperature was 38.5℃, pulse rate was 92 beats/min, blood pressure was 110/68 mmHg, and SpO_2_ was 98% (room air). Physical examination revealed soft, non-tender abdomen without costovertebral angle tenderness. Blood test and urinalysis results are shown in Table 1. Abdominal ultrasonography revealed a 32 × 25 mm nodular shadow on the upper pole of the right kidney (Figure 1). The nodule had irregular margins and indistinct borders, and its interior had a mosaic-like echogenic appearance. Contrast-enhanced computed tomography (CT) of the abdomen revealed a wedge-shaped partially contrast-impaired area in the right kidney (Figure 2). The patient was admitted to the hospital, and tazobactam/piperacillin was administered. No other causes for her fever were found, and AFBN was suspected based on the clinical and radiological findings.

**Table 1:** Laboratory test results RBC: red blood cell, Hb: hemoglobin, PLT: platelet, BUN: blood urea nitrogen, CRP: C-reactive protein, WBC: white blood cell, hpf: high-power field

	On the day of presentation	Reference range
Blood tests		
Leukocyte	10,300/µl	3,300-8,600/µl
Neutrophils	91%	37%-74%
RBC	467 million/µl	386-492 million/µl
Hb	11.9 g/dl	11.6-14.8 g/dl
PLT	256,000/µl	158,000-348,000/µl
BUN	10.8 mg/dl	8.0-20.0 mg/dl
Serum creatinine	0.85 mg/dl	0.46-0.79 mg/dl
CRP	4.66 mg/dl	<0.14 mg/dl
Urinalysis results		
WBC	1-4/hpf	<5/hpf
Ketone	Negative	Negative

**Figure 3:**
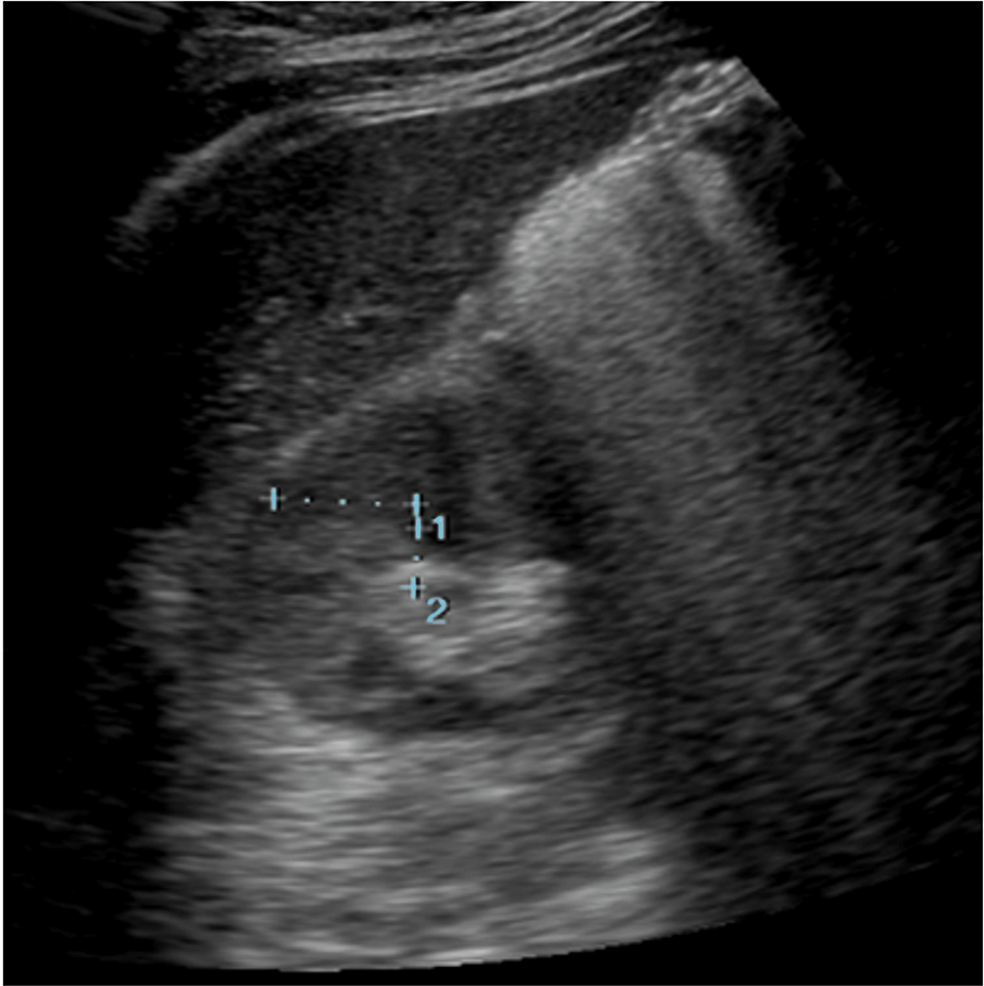
Abdominal ultrasonography showing a shrinking, slightly hypo-absorptive zone in the right kidney (15 × 7 mm) This was four weeks after the first ultrasonography (shown in Figure 1).

Nausea occurred on day 2 of admission and persisted until day 4. The pain extending from the right side of the abdomen to the back had resolved by the time of discharge. Etodolac was continued during hospitalization, and the fever resolved on day 3 of hospitalization. The patient was discharged without drainage on day 7 of hospitalization. Antibiotics were switched to oral amoxicillin and clavulanate that were continued for an additional two weeks. Despite the lack of prior antibiotic administration at the time of presentation, urine and blood cultures were negative. The patient was seen three weeks after discharge from the hospital, and no flare-up of symptoms was noted. Furthermore, blood test results showed that the inflammatory reaction had disappeared. Abdominal ultrasonography showed a shrinking, slightly hypo-absorptive zone in the right kidney (15 × 7 mm) (Figure 3). Since then, the patient has switched her visits to another hospital. Four months have passed since she was discharged from our hospital, with no recurrence of AFBN symptoms and no renal lesions found by ultrasound at the referral hospital. Therefore, we considered the AFBN to be cured, and malignancy was ruled out.

## Discussion

AFBN typically presents as fever (86.13%-98% of cases), lateral abdominal pain (80% of cases), lower urinary tract symptoms (55.89% of cases), and pyuria (75%-97.9% of cases) [1,2,5,6]. Our case was unusual in that it was not accompanied by lower urinary tract symptoms and pyuria. Blood and urine cultures positive for AFBN have been reported in 5%-19% and 59%-91% of cases, respectively [1,2,7]. Despite the absence of prior antibiotic use in our patient, blood and urine cultures did not identify any causative organisms. In cases of pyelonephritis, abdominal contrast CT is recommended when the symptoms persist for 48-72 h after antimicrobial therapy [8]. As our patient presented with fever and abdominal pain without a clear source, CT was performed on admission to determine the cause of the abdominal pain. For cases of idiopathic fever and lateral abdominal or back pain, abdominal ultrasonography or contrast-enhanced CT should be performed, even with negative urine culture results and no pyuria.

The diagnosis of AFBN is important because the duration of antibiotic therapy differs between AFBN and pyelonephritis. An antibiotic treatment for three weeks, rather than two weeks, is considered effective for AFBN in children [7]. For adults, three to four weeks of antimicrobial therapy is considered appropriate; however, there is no consensus. Conversely, the duration of antibiotic therapy for acute pyelonephritis is 5-10 days [9]. Therefore, if treatment is initiated for acute pyelonephritis in the absence of an accurate diagnosis, then the duration of antibiotic therapy will be shorter and the risk of relapse and progression to a renal abscess would be higher [7]. In our patient, AFBN was resolved after three weeks of antibiotic treatment.

The differential diagnosis of renal tumors includes renal malignancy, cyst, renal abscess, and renal infarction. AFBN is characterized by renal hypoperfused wedged-shaped or round and space-occupying lesions with no capsule [2]. These features were also observed in our case. In one case, renal cell carcinoma was suspected initially, and AFBN was diagnosed after nephrectomy [10]. We ruled out malignancy because the wedged-shaped lesion was atypical for renal malignancy and resolved over time. The lesion differed from a cyst or abscess due to the absence of a fluid component and could be differentiated from renal infarction because it did not exhibit the cortical rim sign. Abnormalities can be noted in imaging studies even after symptoms have abated. AFBN lesions frequently persist for 10 days to 4 weeks after onset and almost disappear by weeks 4-8; thereafter, 4.4% of the lesions persist as late renal scaring [2]. Once there is a remission of clinical symptoms, continued treatment is not considered necessary, even if imaging findings remain.

## Conclusions

Acute focal bacterial nephritis is a localized infection of the kidney; its diagnosis changes the duration of antibiotic therapy. For cases involving fever and lateral pain or back pain, and even those without pyuria and with negative urine culture results, it is advisable to perform abdominal ultrasonography and contrast-enhanced CT to differentiate AFBN from other diagnoses, especially pyelonephritis.
